# Effect of Prone Position on Intraocular Pressure in Patients Undergoing Nonocular Surgery: A Systematic Review

**DOI:** 10.7759/cureus.83617

**Published:** 2025-05-06

**Authors:** Eleftheria N Saoulidou, Varvara D Giavi, Nikolaos A Paidakakos, Dimitrios S Papaconstantinou, Antonia N Dimakopoulou, Paraskevi K Matsota

**Affiliations:** 1 2nd Department of Anesthesiology, School of Medicine, National and Kapodistrian University of Athens, Athens, GRC; 2 Anesthesiology, General Hospital of Athens "Georgios Gennimatas", Athens, GRC; 3 Anesthesiology, University General Hospital “Attikon”, Athens, GRC; 4 1st Department of Opthalmology, School of Medicine, National and Kapodistrian University of Athens, Athens, GRC; 5 Opthalmology, General Hospital of Athens "Georgios Gennimatas", Athens, GRC; 6 Neurosurgery, General Hospital of Athens "Georgios Gennimatas", Athens, GRC

**Keywords:** anesthesia, intraocular pressure, postoperative vision loss, prone position, surgery

## Abstract

Postoperative vision loss is a rare complication following surgery in prone positioning. Waking with loss of visual acuity after an elective nonocular surgery is a catastrophic event, not only for the patient but also for the physicians. The specific pathogenesis of postoperative vision loss remains unknown in most cases. This systematic review aims to investigate the effect of prone positioning on intraocular pressure in patients undergoing surgeries and alert physicians to the potential vision loss in the postoperative period. A meticulous research of PubMed, Scopus, and Google Scholar databases was conducted for relevant articles published between January 2012 and September 2023. All relative prospective, retrospective, comparative, and non-comparative studies that investigated prone position and its effect on intraocular pressure during surgery were considered eligible for inclusion in our systematic review. A total of eight studies comprising 649 patients who underwent nonocular surgery under prone positioning were reviewed. Most patients underwent spine surgery with total intravenous anesthesia. The device most frequently used for intraocular pressure measurement was Tono-Pen® XL (Medtronic plc, Galway, Ireland). None of the patients suffered from visual loss postoperatively. Only three patients had vision disturbances after surgery due to prone positioning. There was also a significant rise in intraocular pressure from the supine to the prone position. There is a need to identify crucial factors for the prevention of postoperative vision loss.

## Introduction and background

Intraocular pressure (IOP) is essential to maintain the refractive properties of the eye and is defined as the fluid pressure inside the eye. An increase in IOP reduces the perfusion of the ocular structures in a linear manner, and at elevated levels, it is more important than blood pressure in determining retinal function [1]. Spine surgical procedures are associated with a 10-fold increased risk of retinal injury as they are associated with an increase in IOP [2,3]. The incidence of postoperative vision loss after spine surgery ranges from 0.013% to 1%, with the most frequently quoted risk being 0.2%, especially during procedures involving prone positioning [4-7]. 

Although vision loss is a rare complication, it is a devastating one. The specific pathogenesis of perioperative vision loss remains elusive in most cases [8]. There are three main types of postoperative vision loss: (i) central retinal artery occlusion, typically occurring from direct pressure on the globe of the eye, (ii) cortical blindness, resulting from an infarct of the occipital area of the brain, and (iii) ischemic optic neuropathy, resulting from inadequate perfusion of the optic nerve [5]. There are multiple factors contributing to postoperative vision loss, including prolonged operative times, long‐segment spinal instrumentation, anemia, intraoperative hypotension, diabetes, obesity, male sex, the Wilson frame, greater estimated blood loss, microvascular pathology, and high volume fluid replacement [6,9].

The aim of this study is to review the current literature on the effect of prone positioning on IOP in patients undergoing nonocular surgery.

## Review

Materials and methods

The present systematic review was designed in accordance with the guidelines for the Preferred Reporting Items for Systematic Reviews and Meta-Analyses (PRISMA) based on the authors’ pre-determined eligibility criteria. We performed an independent and meticulous search of the literature, excluded overlaps, and tabulated the selected indices in structured forms. 

Inclusion and Exclusion Criteria

Only articles published in English were included. All appropriate prospective, retrospective, comparative, and non-comparative studies that investigated the effect of the prone position on IOP in patients undergoing nonocular surgery were considered eligible for inclusion. Only studies in which the minimum age of study participants was 18 years or older were included. Reviews, case reports, and animal studies were excluded from tabulation and analysis. Studies were also excluded if data from only one time point of IOP measurement were present or the IOP was not formally screened. 

Database Search

**Figure 1:**
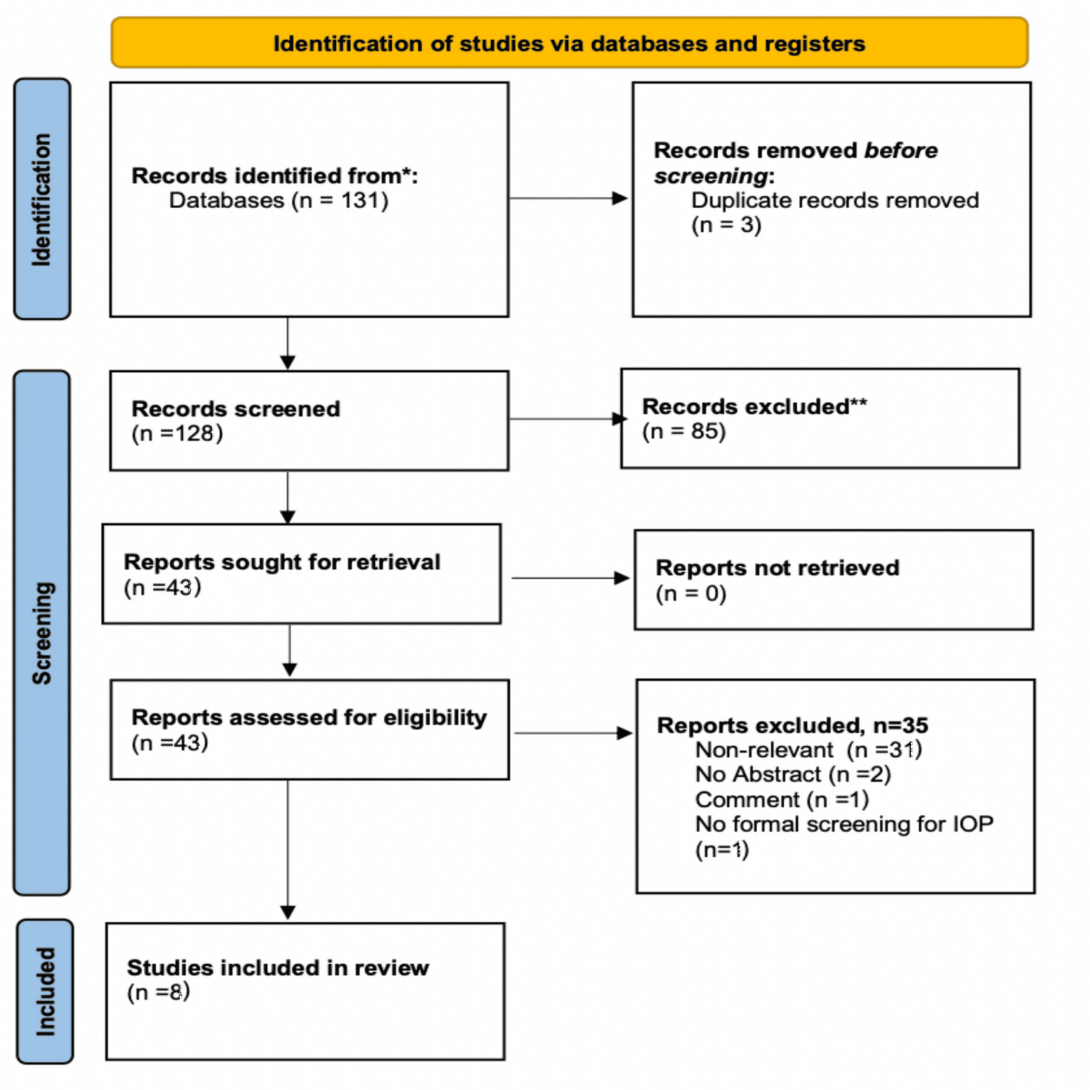
PRISMA flow chart showing the selection of studies * PubMed, Scopus, and Google Scholar databases ** Articles published before 2012: 66; Articles not published in English: 2; Articles with human participants: 14; Systematic reviews: 3 PRISMA: Preferred Reporting Items for Systematic Reviews and Meta-Analyses

We systematically searched for articles published between January 2012 and September 2023 using PubMed, Scopus, and Google Scholar databases, along with the references of the articles that were retrieved in full text. The following keywords were used for the search: “prone position”, "surgery”, “intraocular pressure”, “postoperative vision loss”. A minimum number of search keywords was utilized in an attempt to assess an eligible number that could be easily searched while simultaneously minimizing the potential loss of articles. Articles that fulfilled or were deemed to fulfill the inclusion criteria were retrieved. The PRISMA flow diagram schematically presents the stages of article selection (Figure 1).

Quality Assessment

The risk of bias in the included randomized controlled clinical trials (RCTs) was independently assessed by two reviewers (ENS and PKM) using the Cochrane's tool for assessing the risk of bias in randomized trials (ROB-2). The domains assessed included the randomization process, deviations from intended interventions, missing outcome data, measurement of outcome, selection of the reported results, and other biases.

Results

The initial search revealed 131 studies. Three articles were rejected because they were systematic reviews, 66 articles were excluded because they were published prior to 2012, two were excluded because the language was not English, and 14 non-human subject studies were excluded. The full text of 43 articles was evaluated, and 35 articles were omitted because they did not meet the inclusion criteria. The flowchart of selected studies with specific reasons for exclusion is shown in Figure 1. A total of eight studies, published between January 2012 and September 2023, with 649 patients, were eligible and were included in the analysis.

Type of Surgery

The majority of participants underwent spine surgery (n=349), while others underwent intracranial procedures (n=32), percutaneous nephrolithotomy (n=45), gynecological surgery (n=42), colorectal surgery (n=34), thoracic surgery (n=47), urology surgery (n=49), general surgery (n=24), breast surgery (n=8), cardiac surgery (n=10), and lower limp arthroscopy (n=9).

Type of Anesthesia

Most patients received general anesthesia either with sevoflurane (n=129) or total intravenous anesthesia (TIVA) with propofol infusion (n=451). Some patients were under spinal anesthesia (n=50). The study of Carey et al. [2] does not mention the anesthesia protocol used in their 19 patients.

Method of IOP Measurement

Tono-Pen® XL (Medtronic plc, Galway, Ireland) was the device most frequently used for IOP measurements, but there were studies where different methods have also been used. The Perkins Tonometer MK2 (Clement Clarke International Ltd, Mountain Ash, Wales, United Kingdom) was used in Deniz et al.'s study [10], while Pinar et al. used the Tono-Pen AVIA® Handheld Tonometer (Reichert Technologies, Depew, New York, United States) [11]. Sun et al. used the Icare PRO TA03 Tonometer (Icare Finland Oy, Vantaa, Finland) for IOP measurement [12]. In Lyzohub et al.'s study, IOP was measured with the Maklakov Method [13].

Head Positioning

Sugata and his co-investigators used the LT-700 spinal frame (ISO Medical Systems Inc., Tokyo, Japan) with a headrest [3], while in Carey et al.'s study, the head was secured using a Mayfield head attachment to facilitate tonometric measurements [2]. In the randomized trial of Deniz et al., the head was placed on a silicone headrest without external direct compression to both eyes [10]. In Pinar et al.'s trial [11] and Sun et al.'s study [12], a horseshoe-shaped gel ring was used. The other studies did not mention the method used to secure the head during the prone position.

Postoperative Complications

Most of the studies report that they had no patients with visual loss or visual disturbances either intraoperatively or postoperatively. Emery et al. noted that one patient sustained a mild corneal abrasion that resolved in 24 hours with the use of ophthalmic ointment [8]. Similarly, Carey et al. in their study observed that one patient experienced a corneal abrasion with no other reported ophthalmic complications [2]. In Czorlich et al.'s trial, the postoperative ophthalmological examination showed one corneal irritation in a single eye requiring no further treatment [14].

Assessment of Risk of Bias

**Figure 2:**
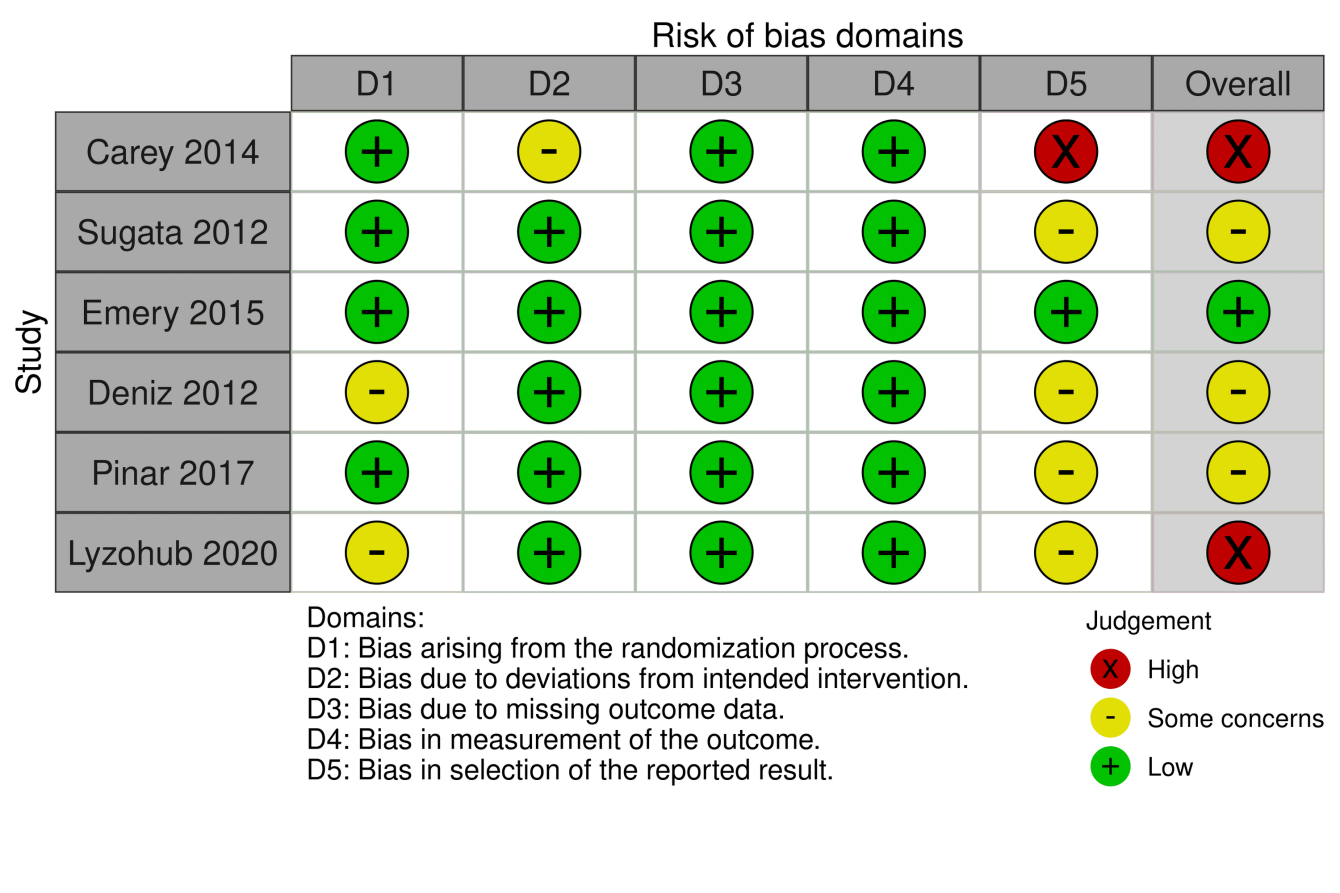
Risk of bias summary References: Carey et al., 2014 [2], Sugata et al., 2012 [3], Emery et al., 2015 [8], Deniz et al., 2012 [10], Pinar et al., 2017 [11], Lyzohub et al., 2020 [13]

Out of the studies included in our analysis, most reported some concerns in terms of the selection of the reported results. The study of Emery et al. showed an overall low risk of bias [8], while the studies of Sugata et al. [3], Deniz et al. [10], and Pinar et al. [11] showed some concerns as far as the risk of bias is concerned. The studies of Carey et al. [2] and Lyzohub et al. [13] showed high risk of bias (Figure 2).

 The summary and key characteristics of the included studies are given in Table 1.

**Table 1:** Studies included in the systematic review IOP, intraocular pressure; IOP3, Intraocular pressure at the 10th minute of prone position placement; IOP4, intraocular pressure when the operation was over; TIVA, total intravenous anaesthesia; CSF, cerebrospinal fluid

Study	Year	Study design	Type of Surgery	Sample	Groups	IOP measurement	Results/ p value
Carey et al. [2]	2013	Prospective randomized control study	Prone spine surgery	n=19	Neutral position (7) Five degree (5^o^) reverse Trendelenburg (6) Ten degree (10^0^) reverse Trendelenburg (6)	Tono-pen XL	10^o ^reverse Trendelenburg found to be superior to a 5^o^ reverse Trendelenburg (no determination of statistical significance)
Sugata et al. [3]	2012	Prospective randomized control trial	Prone spine surgery	n=24	Propofol (12) Sevoflurane (12)	Tonopen XL hand held tonometer	No significant effect on IOP between groups
Emery et al. [8]	2015	Randomized prospective trial	Lumbar spine fusion	n=52	Head in neutral position (27) Head elevated 10^o^ (25)	Applanation tonometer (Tono-pen)	IOP lower by elevating the head 10^o^ (p=0.0074)
Deniz et al. [10]	2012	Prospective randomized control trial	Prercutaneous nephrolithotomy	n=45	Neutral position (23) Prone with their heads rotated 45^o^laterally to the right side (22)	Clement Clarke Perkins Tonometer MK2	IOP in the upper positioned eye was lower (p<0.05)
Pinar et al. [11]	2018	Prospective randomized control trial	Lumbar disc surgery	n=40	Spinal anesthesia (20) General anesthesia (20)	Tono-pen AVIA	IOP increase was lower in patients under spinal anesthesia (p=0.018 in IOP3 and P<0.001 in IOP4)
Sun et al. [12]	2022	Cross-sectional observational study	Gynecological surgery, spine surgery, colorectal surgery, thoracic surgery, urology surgery general surgery, breast surgery cardiac surgery and lower limp arthroscopy	n=325	Lithotomy position (76) Lateral position (96) Prone position (102) Supine position (51)	Icare TA03	Different change patterns with the surgical process and position change Prone position (p<0.01)
Lyzohub et al. [13]	2020	Randomized prospective trial	Spine surgery	n=80	Spinal anesthesia (30) TIVA anesthesia 45^o^head rotation (25) TIVA no head rotation (25	Maklakov Method	IOP in the upper position Eye was lower in TIVA (p<0.05) No difference between spinal and TIVA group
Czorlich et al. [14]	2019	Prospective Observational Study	Spine surgery in prone and intracranial procedures	n=64	Spine surgery in prone position (16) Intracranial procedures in prone position (16) Intracranial pathologies in a modified lateral position (16) Spine surgeries in prone position with an intact dura (16)	Tono-pen XL	Opening the dura mater with CSF loss was associated with decrease in IOP (p<0.0007)

Discussion

This systematic review aimed to assess the effect of the prone position on IOP in patients undergoing nonocular surgery. Sugata et al. investigated the effect of prone position on IOP in patients undergoing spine surgery under general anesthesia, with either TIVA or sevoflurane and showed that after positioning patients in prone position, IOP values were significant higher in both groups regardless of the anesthetic agents used; specifically, the rise was in the Propofol group from 8.9±3.5 to 21.9±5 mmHg and in the sevoflurane group from 11.6±3.9 to 24.8±3.4 mmHg, respectively [3]. 

In Emery et al.'s study, different head positions were investigated in patients who underwent lumbar spine fusion [8]. Initial IOP measurements obtained with the patient prone changed in Group I, in which the patients were in neutral position, from 14.24±4.96 to 23.96±4.93 mmHg, and in Group II, where the head was elevated such that the face was at 10^o^ angle in relation to the horizontal plane, from 13.98±4.82 to 22.21±3.87 mmHg. Czorlich et al., who studied the effect of prone position in different types of surgery, showed that IOP was raised in spine surgery (Group A) from 10.5±3.5 to 23.0±5.2 mmHg, in intracranial procedures (Group B) from 12.4±3.5 to 24.1±3.3 mmHg and in spine surgeries with an intact dura (group D) from 11.7±4.1 to 23.6±6.0 mmHg [14]. Pinar et al., who investigated the effect of prone position on IOP under different types of anesthesia, found that in patients who received general anesthesia, the IOP changed from 19.7±4.1 to 21.6±3.1 mmHg, while in those under spinal anesthesia, a slight increase from 18.4±1.9 to 19.3±2.7 mmHg was observed [11]. In Lyzohub et al.'s study, in patients who received spinal anesthesia (Group I), IOP raised from 15.9±1.0 mmHg before surgery to 17.2±1.2 mmHg after surgery, while in patients who recieved general anesthesia with TIVA, it was found that, according to the head rotation (Group IIa and Group IIb), in Group IIa, where the head was placed with 45^o^ rotation, IOP raised from 16.1±1.4 to 18.4±1,4 mmHg and in Group IIb with no head rotation, IOP changed from 15.9±1.4 to 18.8±1.7 mmHg after the completion of surgery [13]. The remaining studies only mention that IOP was significantly higher in the prone position compared to other positions, suggesting that the prone position may have a greater effect on IOP than other positions [2,12].

In spite of the rise of IOP in the prone position, no incidence of postoperative vision loss was seen. This result might be attributed to the fact that there is autoregulation of the blood flow of the optic nerve [15]. When the rise in IOP critically reduces ocular perfusion pressure beyond autoregulation limits, ocular blood flow becomes compromised, leading to retinal dysfunction [16]. Pillunat et al. showed in their study that in healthy subjects, the optic nerve blood flow is typically held nearly constant despite IOP elevation [15]. The critical point where blood flow declines most prominently is IOP values above 45 mmHg. In 1986, Riva et al. conducted a study demonstrating that retinal autoregulation is up to IOP between 27 and 30 mmHg [17]. However, in some individuals, autoregulation seems to be absent [15].

Increased IOP may also contribute to other ophthalmological complications, including central retinal occlusion, retinal ischemic injury, and deterioration of glaucoma [18].

In this systematic review, we demonstrated that in all included studies, IOP increased in the prone position, so some measures should be undertaken intraoperatively to prevent or minimize this effect and simultaneously to reduce the risk of ophthalmological complications. Emery et al. [8], as well as Carey et al. [2], have shown that patient positioning in a 5-10^o^ reverse Trendelenburg prone position may be an intervention to lower IOP. Even the choice of anesthesia may affect the IOP intraoperatively. Pinar et al., in their clinical trial, demonstrated that the IOP increase was lower in patients under spinal anesthesia [11]. For all the above reasons, IOP measurements during spinal surgery in the prone position should be advisable.

There are several limitations in this study. First of all, the number of studies was very small (n=8), and some involved a small number of patients. Furthermore, there are no specific time settings for the measurements of IOP, which means that there was a lot of differentiation in the measurements. Some studies had no documentation of IOP in the prone position but only reported measurements of IOP in the supine position before and after the completion of surgery and derived their results based on these measurements alone. Finally, the review protocol has not been registered at PROSPERO (International Prospective Register of Systematic Reviews).

## Conclusions

While no cases of postoperative vision loss were observed, it has been found that IOP increases significantly during the prone position surgery. There should be some strategies to prevent this increase, including the type of anesthesia, the position of the surgical table, and the position of the head on the table. Further studies are required regarding the increase of IOP in patients undergoing surgery in the prone position in order to find and implement interventions to minimize the risk of postoperative vision loss and other ophthalmologic complications. 
